# Comparative analysis of the extent of protein–protein interactions in icosahedral viral capsids

**DOI:** 10.1002/pro.70257

**Published:** 2025-08-25

**Authors:** Noah J. Zimmerman, Oscar Rojas Labra, Vijay S. Reddy

**Affiliations:** ^1^ The Hormel Institute University of Minnesota Austin Minnesota USA; ^2^ Department of Genetics, Cell Biology and Development University of Minnesota Minneapolis Minnesota USA; ^3^ Department of Integrative Structural and Computational Biology The Scripps Research Institute La Jolla California USA

**Keywords:** buried surface area, coat proteins, icosahedral viruses, protein–protein interactions, *T*‐number, viral capsids

## Abstract

Nonenveloped viruses package, carry, and deliver their genomes to the targeted cells using protein shells known as capsids. The viral capsids come in different shapes and sizes, most exhibiting helical or icosahedral symmetries. Here, we analyzed 634 icosahedral capsids at high resolution (<4 Å) from 39 virus families with *T*‐numbers ranging from 1 to 9 and evaluated the aggregated buried surface areas (BSAs) at the unique interfaces as a measure of capsid strength and protein–protein interactions (PPIs). The BSAs were further analyzed relative to their capsid diameters and the calculated molecular weight (MW) of coat protein subunits (CPs) occupying the icosahedral asymmetric unit (IAU). Our results show that naturally occurring viral capsids exhibit stronger PPIs relative to non‐native and/or engineered capsids. Interestingly, the “*T* = 2” capsids cluster distinctly, exhibiting weaker PPIs relative to their capsid size and subunit MWs. Furthermore, the normalized BSAs by the MW of the CPs present in the IAU are fairly constant across different capsids, suggesting that the extent of the PPIs is proportional to the CP size with a few exceptions (e.g., “*T* = 2” capsids). We also identified the range of capsid diameters and MWs of CPs forming different *T* = number capsids, which suggest a CP of 30–50 kDa can be used to build any quasi‐equivalent capsid with *T*‐numbers 1–9. Furthermore, we identified the strongest capsids available at various diameters at 25 Å intervals. Taken together, in addition to the targeting specificities, the results from this study are useful for choosing viral capsids for biomedical applications.

## 1 INTRODUCTION

**FIGURE 1:**
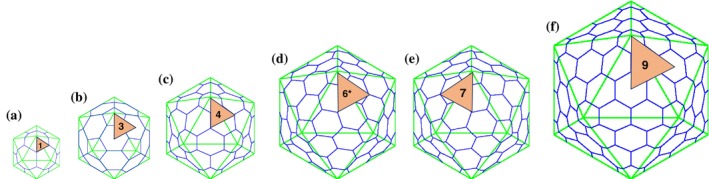
Schematic diagrams of icosahedral lattices of the capsids described in this study and are drawn on a relative scale. (a) *T* = 1 icosahedral lattice (*h* = 1, *k* = 0) with an icosahedral asymmetric unit (IAU) identified by a beige color triangle. The number in the triangle indicates the number of subunits occupying the IAU. The “*T* = 2” capsid is essentially a *T* = 1 capsid with two subunits occupying the IAU. (b) *T* = 3 icosahedral lattice (*h* = 1, *k* = 1). (c) *T* = 4 icosahedral lattice (*h* = 2, *k* = 0). (d) *T* = 7*d* icosahedral lattice (*h* = 1, *k* = 2). Of note, all the capsids that display *T* = 7*d* lattice to date are composed of only the subunit pentamers, and as a result contain only six subunits in the IAU, while the *T* = 7*l* capsids contain seven subunits in the IAU. (e) *T* = 7*l* icosahedral lattice (*h* = 2, *k* = 1). (f) *T* = 9 icosahedral lattice (*h* = 3, *k* = 0). The icosahedral lattices shown in the figure were obtained from the Icosahedral Server utility available at VIPERdb (https://viperdb.org/Icosahedral_Server.php) (Carrillo‐Tripp et al., 2009; Montiel‐Garcia et al., 2021).

Nonenveloped viruses (NEVs) are mainly composed of two components—viral coat (capsid) and genome (DNA or RNA) (Fields, 1996; Gelderblom, 1996). Viral capsids are protein shells comprising multiple copies of one or a few capsid proteins (CPs) assembled to encapsulate, protect, and deliver the viral genome to the susceptible cells (Mateu, 2013). Unlike NEVs, enveloped viruses contain a host‐derived lipid membrane with envelope glycoproteins embedded in them. Most NEV capsids display helical, filamentous, or icosahedral architectures assembled from CPs exhibiting a limited number of tertiary structures (e.g., jelly roll beta‐barrel) (Harrison et al., 1996; Prasad & Schmid, 2012; Rossmann & Johnson, 1989). The simplest of icosahedral capsids are composed of 60 copies of a single CP (gene product) that occupies an icosahedral asymmetric unit (IAU, 1/60th of an icosahedron) (Crick & Watson, 1956). Larger capsids contain multiple copies of CP occupying the IAU (Figure 1) as determined by the triangulation (*T*) number according to the quasi‐equivalence theory of Caspar and Klug (Caspar & Klug, 1962). Only certain *T*‐numbers are permitted according to the formula *T* = *h* ^2^ + *hk* + *k* ^2^, where *h* and *k* are integers, for example *T* = 1, 3, 4, 7, 9, etc. (Caspar & Klug, 1962; Johnson & Speir, 1997). A *T* = 3 capsid contains three copies of a CP in the IAU, and a total of 180 CPs are assembled into an icosahedral capsid. However, there are exceptions to the quasi‐equivalence theory. For example, the inner capsids of Bluetongue and Reovirus cores (Grimes et al., 1998; Reinisch et al., 2000) and the capsids from Totiviridae and Picobirnaviridae families contain two copies of the respective CPs in an IAU, resulting in “*T* = 2” capsids containing 120 subunits (Duquerroy et al., 2009; Naitow et al., 2002). However, technically speaking, the *T* = 2 architecture is not formally permitted according to the above *T*‐number formula; hence, they are empirically referred to here as “*T* = 2” capsids. Furthermore, *T* = 7*d* capsids of Polyomaviridae feature exclusively CP pentamers without any hexamers and hence are assembled from 72 pentamers (360 copies) of a CP as opposed to 420 subunits expected in a *T* = 7 capsid (Goetschius et al., 2021; Liddington et al., 1991; Stehle et al., 1996; Stehle & Harrison, 1996). Despite these exceptions to the definition of *T*‐number, it has been useful and commonly used in virology for describing various icosahedral capsid architectures.

It is generally accepted that the Buried Surface Areas (BSAs) between protein subunits reflect the extent of protein–protein interactions (PPIs) bonding them (Eisenberg & McLachlan, 1986; Horton & Lewis, 1992; Janin, 1995; Reddy et al., 1998). Previous studies analyzed the PPI in viral capsids involved a limited number of them because of the availability of fewer high resolution capsid structures (Bahadur et al., 2007; Damodaran et al., 2002; Shepherd & Reddy, 2005). In this study, we analyzed 634 NEV capsids from 39 families displaying eight different capsid architectures (*T* = 1, “2”, 3, 4, 7*d*, 7*l*, 9 and *pseudo*‐*T* = 3). We used the aggregated BSAs at the unique CP subunit interfaces in each capsid as an estimate of the extent of the PPIs to assess the strength of viral capsids relative to each other as a function of capsid diameters and molecular weight of CPs occupying the IAU.

## 2 RESULTS AND DISCUSSION

In addition to natively occurring viral capsids grouped according to the respective virus families, we also included non‐native and synthetic capsids in this analysis. The following terminology is used to distinguish different types of capsids included in this study. The “non‐native” capsids are those with different *T*‐numbered architectures (e.g., *T* = 1) distinct from the natively occurring capsids (e.g., *T* = 3). The non‐native capsids are formed either due to N‐terminal truncations of the WT‐CPs (e.g., Brome mosaic virus, Norovirus) or formed by one of the major capsid proteins (e.g., Penton base of adenoviruses) when overexpressed. The “synthetic” capsids are formed either from the de novo designed CPs (e.g., PDBs: 8f53, 7b3y, 5im4) or naturally occurring non‐viral capsids (e.g., Encapsulins, Pyruvate dehydrogenase cores). Notably, the synthetic capsids are grouped into a *pseudo* virus family called Nanoparticles. The “altered” capsids are those with missing VP4 peptide in the Picornaviridae family.

### 2.1 *T* = 1 capsids

**FIGURE 2:**
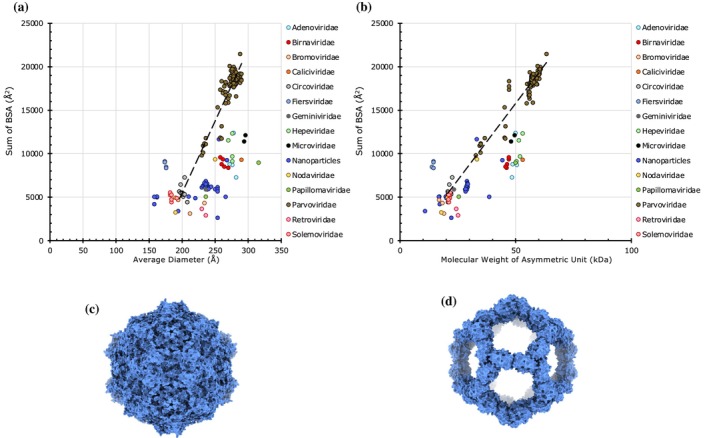
The magnitude of protein–protein interactions (PPIs) in *T* = 1 capsids. (a) A plot of cumulative sums of BSAs at the unique CP interfaces as a function of average diameter of the *T* = 1 capsids (*n* = 213). The capsids are grouped by family as indicated in the legend on the right. The points below the trendline indicate weaker PPIs than expected according to their capsid size, while those above the trendline suggest stronger interactions. The trendline was calculated based on native capsids from Circoviridae and Parvoviridae. (b) A plot of cumulative sums of BSAs as a function of the molecular weight (MW) of the subunit occupying the asymmetric unit (*n* = 213). Capsids are grouped by family as in panel A. (c) Surface representation of an example Parvovirus capsid (PDB: 8EP2), known to have strong PPIs (cumulative BSAs: 21,454 Å^2^). (d) Surface representation of an example synthetic nanoparticle (PDB: 7B3Y) that exhibits very weak PPIs (cumulative BSAs: 2610 Å^2^).

*T* = 1 capsids, with *h* = 1, *k* = 0 indices, are the simplest protein shells formed by the spherical viruses, composed of 60 copies of a single type of CP with one subunit occupying an IAU. We analyzed 213 *T* = 1 capsids from 15 virus families, with diameters ranging from 158 to 316 Å, while the BSAs varied between 2610 and 21,454 Å^2^ (Figures 2 and S1). Table S1 shows the PDB‐IDs of various *T* = 1 capsids along with the others considered in this report.

The only native *T* = 1 viral capsids in the dataset belong to the viruses from the Circoviridae and Parvoviridae families. We plotted the cumulative sum of BSAs at the unique CP–CP interfaces as a function of the capsid diameters and the MW of the CPs occupying the IAU and calculated the trendline based on the data from these native capsids (Figure 2a,b). The strongest PPIs observed among the *T* = 1 capsids belong to the Parvoviridae family, with the majority having BSAs in the range of 15,000–20,000 Å^2^ (Figure 2a,c). However, we observed two clusters of Parvoviridae capsids, with the bulk of them having BSAs ~19,000 Å^2^, and a smaller cluster with BSAs around 11,000 Å^2^. The latter cluster belongs to the invertebrate‐targeting Densovirus genus (Figure S2) (Kaufmann et al., 2011). Not surprisingly, the capsids from the Circoviridae family showed weaker PPIs consistent with the smaller size of CPs (21–22 kDa) and correspondingly smaller diameters (196–208 Å) compared to Parvoviridae CPs (32–63 kDa) displaying relatively larger diameters (230–290 Å).

Significantly, most non‐native and synthetic *T* = 1 capsids fall below the trendline, except for the capsids from Fiersviridae (previously Leviviridae), which typically form *T* = 3 capsids, showing significantly higher BSAs corresponding to the size of the capsid (Figure 2a,b). The non‐native viral capsids that fall below the trendline are those formed by the modified CPs (e.g., Hepeviridae, Papillomaviridae) and the penton base of adenoviruses (Figure 2a). The majority of the non‐viral protein shells (e.g., bacterial encapsulins) and synthetic/engineered capsids that are grouped into a family of “Nanoparticles” cluster below the trendline, implying weaker PPIs corresponding to their capsid size. Some of these engineered capsids are porous with big holes in them (e.g., TIP60) (Obata et al., 2021), thereby resulting in weaker PPIs (Figure 2d). Interestingly, the plot of BSAs versus molecular weights shows similar trends as in BSAs versus diameters but with much sharper clustering of respective families (Figure 2b).

### 2.2 “*T* = 2” capsids

**FIGURE 3:**
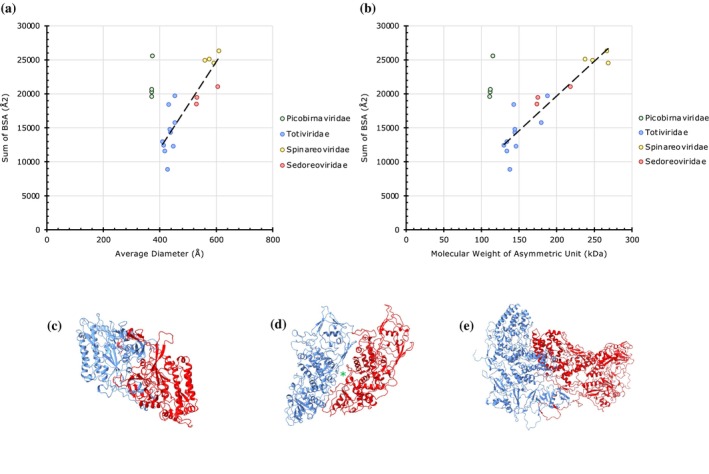
The extent of protein–protein interactions (PPIs) in “*T* = 2” capsids. (a) A plot of cumulative sums of BSAs at the unique CP interfaces as a function of the average diameter of “*T* = 2” capsids (*n* = 21). The capsids are grouped by family as indicated in the legend on the right. (b) A plot of cumulative sums of BSAs as a function of the aggregate molecular weight of CPs in the asymmetric units of “*T* = 2” capsids (*n* = 21). (c) Arrangement of CPs in the IAU of a Picobirnaviridae capsid (PDB: 2VF1) with strong PPIs at the intra‐subunit interface (BSA: 13,034 Å^2^). (d) Arrangement of CPs in the IAU of a Totiviridae capsid (PDB: 7M12) that exhibit weak PPIs at the intra‐subunit interface (BSA: 3604 Å^2^) The asterisk points to the gap that exists between the CPs in support of weaker PPIs. (e) Arrangement of CPs in the IAU of the inner shell of a Reoviridae capsid (PDB: 3JAY) that display moderate PPIs at the intra‐subunit interface (BSA: 7096 Å^2^).

Strictly speaking, “*T* = 2” capsids are not allowed according to the formula of *T*‐number (*T* = *h* ^*2*^ *+ hk + k* ^*2*^, where *h* and *k* are integers), as defined by Caspar and Klug (1962). However, several instances of capsids composed of 120 CPs are observed in nature, with two copies of a CP occupying the IAU, referred to here as “*T* = 2” capsids (e.g., capsids from the Totiviridae family). We analyzed 21 “*T* = 2” capsids from four viral families with diameters ranging from 372 to 610 Å and the BSAs from 8891 to 26,311 Å^2^ (Figure 3).

The capsids from Picobirnaviridae show strong PPIs (BSAs: 19,605–25,586 Å^2^) despite having smaller diameters compared to others (~372 Å) (Figure 3a,b) (Duquerroy et al., 2009). This is due to a strongly intertwined interface (BSA: 13,034 Å^2^ in Picobirnavirus, PDB: 2VF1) between the subunits that occupy the IAU compared to similar interfaces in other capsids (BSA: 7096 Å^2^, in a Cypovirus *T* = 2 capsid, PDB: 3JAY) (Figure 3c,e) (Yu et al., 2015). Interestingly, the two clusters of Picobirnaviridae seen in Figure 3a,b belong to viruses infecting human (lower) and rabbit (upper) hosts. The rabbit Picobirnavirus displays longer and extended loops at the above intra‐IAU interface compared to the shorter loops with compact conformations seen in its human counterpart, thereby resulting in weaker PPIs in the latter (BSAs: 13,034 Å^2^ vs. 10,890 Å^2^) (Figure S3) (Duquerroy et al., 2009; Ortega‐Esteban et al., 2020). There is also a distinct clustering observed among the two Reoviridae subfamilies, with the lower BSA group being composed solely of Sedoreoviridae (e.g., Bluetongue virus) (Grimes et al., 1998) and the higher BSA group being composed of Spinareoviridae (e.g., Reovirus) (Reinisch et al., 2000).

Notably, despite having a larger diameter and subunit size (MW) than Picobirnaviridae, the capsids of the Totiviridae family exhibit the lowest cumulative BSAs among the “*T* = 2” capsids (Naitow et al., 2002). This is due to weak interactions observed between the CPs occupying the IAU, especially when compared to the other “*T* = 2” IAUs (Figure 3c–e). These reduced interactions likely result in weaker capsids of the Totiviridae family.

### 2.3 *T* = 3 capsids

**FIGURE 4:**
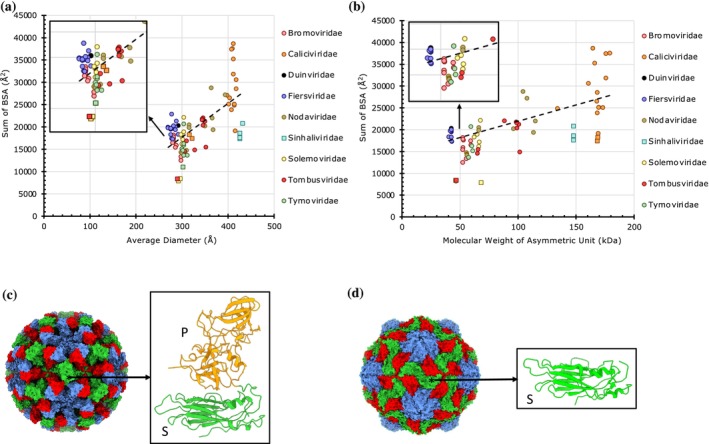
The magnitude of protein–protein interactions in *T* = 3 capsids. (a) A plot of cumulative sums of BSAs at the unique CP interfaces as a function of the average diameter of *T* = 3 capsids (*n* = 89). Capsids are grouped by family as indicated in the legend on the right. (b) A plot of cumulative sums of BSAs as a function of the aggregate molecular weight of CPs in the asymmetric units of *T* = 3 capsids (*n* = 89). (c) Surface representation of an example Calicivirus (Norwalk virus) capsid (PDB: 1IHM) is known to have strong PPIs (cumulative BSAs: 27,048 Å^2^). Shown on the right is the tertiary structure of a Norovirus CP composed of shell (S) and protruding (P) domains. (d) Surface representation of a mouse norovirus capsid (PDB: 6P4L) consists of CPs comprising only S‐domain (right) that exhibit weak PPIs (cumulative BSAs: 17,407Å^2^).

With three copies of a CP (single gene product) occupying the IAU, the *T* = 3 capsids (*h* = 1, *k* = 1) are composed of 180 CPs. We analyzed a total of 89 *T* = 3 capsids from nine viral families with diameters in the range of 270–432 Å, and the BSAs varied between 8375 and 38,654 Å^2^ (Figure 4). The trendline shows that, generally, larger diameter capsids display higher BSAs (Figure 4a). However, the exceptions are Fiersiviridae (formerly Leviviridae) (Persson et al., 2008) and Sinhaliviridae (Chen et al., 2023), which fall above and below the trendline, implying stronger and weaker interactions, respectively (Figure 4a).

The capsids from Caliciviridae show an especially wide range of BSAs for the same capsid sizes, with some being twice as high as others in the same family. The Caliciviridae capsids that show lower BSAs are lacking the P (protruding) domain of the CP (e.g., PDB: 6P4L) (Sherman et al., 2019), while those with higher BSAs contain both the S (shell) and P domains (e.g., PDB: 1IHM) (Prasad et al., 1999), hence form stronger capsids (Figure 4). The *T* = 3 capsids of Sinhaliviridae, which also form *T* = 4 capsids, show lower BSAs compared to the other *T* = 3 capsids with similar diameters. It is noteworthy that we omitted the *T* = 3 envelope (E) glycoprotein shells of Flaviviridae from the analysis as they are not technically viral capsids, and also each subunit is composed of multiple chains (e.g., envelope and matrix proteins) (Lim et al., 2021). A handful of capsids with weaker interactions belong to the genera Polerovirus and Leteovirus in the Solemoviridae and Tombusviridae families, respectively (Figure 4a,b) (Byrne et al., 2019).

### 2.4 *Pseudo*‐*T* = 3 capsids

**FIGURE 5:**
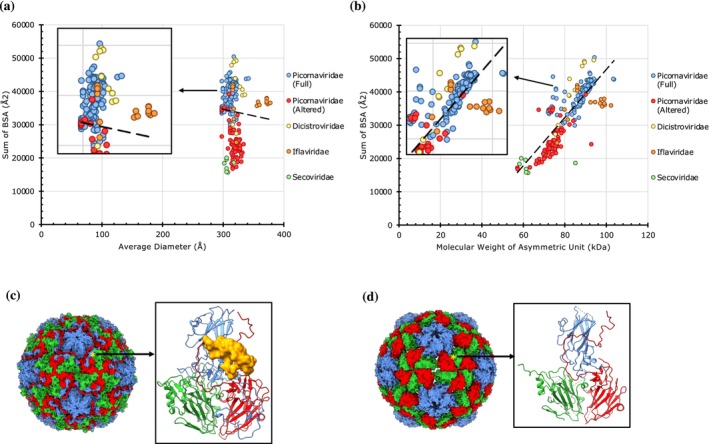
The extent of protein–protein interactions in *pseudo*‐*T* = 3 capsids. (a) A plot of cumulative sums of BSAs at the unique CP interfaces as a function of average diameter in *pT* = 3 capsids (*n* = 241). Capsids are grouped by family as indicated in the legend on the right. (b) A plot of cumulative sums of BSAs as a function of aggregate molecular weight of CPs in the IAU of *pT* = 3 capsids (*n* = 241). (c) Surface representation of human rhinovirus‐2 (PDB: 1FPN), an example full Picornavirus capsid, which exhibits strong PPIs (cumulative BSAs: 35,488 Å^2^). Shown on the right is a protomer (ribbon representation), a view from inside of the capsid. VP4 is shown in the surface representation. (d) A representative structure of an altered Picornavirus capsid, empty rhinovirus 14 particle (PDB: 7NUM) that exhibits weak PPIs (cumulative BSAs: 16,733 Å^2^). Shown on the right is a protomer (ribbon representation), a view from the center of the capsid. VP4 is absent in the altered capsids.

Unlike the *T* = 3 capsids, where three copies of chemically identical subunits occupy an IAU, *pseudo*‐*T* = 3 (*pT* = 3) capsids contain three chemically non‐identical subunits occupying the IAU (e.g., VP1, VP2, VP3 of Picornaviridae) (Filman et al., 1989; Rossmann & Johnson, 1989). We analyzed 241 *pT* = 3 capsids from four viral families, whose diameters ranged from 298 to 378 Å, and the BSAs varied between 15,621 and 50,340 Å^2^ (Figure 5). The majority of *pT* = 3 capsids arise from the Picornaviridae family, and these capsids cluster vertically along the *Y*‐axis into two groups (Figure 5a)—full (blue circles) and altered (red circles) capsids. Of note, the full capsids contain an extra polypeptide chain, VP4, on the interior of the capsid that ties all the (VP1–VP3) subunits together, akin to a “molecular strap”, thereby resulting in higher BSAs and overall strengthening the capsid (Figures 5c,d, S4). The altered capsids, which represent intermediates in the picornavirus infection cycle, lack the VP4 chain (Buttner et al., 2022; Yang et al., 2022). The loss of VP4 results in lower BSAs, thereby rendering the altered capsids weaker. In support of the above discussion, the full capsids from Picornaviridae show higher BSAs (29,862–50,340 Å^2^), while the altered capsids exhibit lower BSAs (16,733–39,979 Å^2^). There appears to be a slight overlap between the two clusters. Particularly, both clusters display similar diameters (298–334 Å). The full and altered capsids from Dicistroviridae (Tate et al., 1999) cluster similarly with those from the Picornaviridae family (Figure 5a).

Furthermore, the *pT* = 3 capsids also cluster into two groups horizontally, along the *X*‐axis representing the diameters. One cluster, with diameters around 320 Å, contains capsids of Picornaviridae and Dicistroviridae, while the second cluster, having larger diameters between 356 and 378 Å, is composed of capsids from the Iflaviridae family comprising honeybee viruses. The larger‐size Iflaviridae capsids (e.g., PDB: 5J96) display an extra domain that is part of the VP3 subunit and projected out on the outer surface, leading to larger capsid size (Figure S5) (Kalynych et al., 2016). However, interestingly, this domain is not involved in any PPIs as observed between the P‐domains in Caliciviridae (Prasad et al., 1999).

The members of Secoviridae, known as “plant Picornaviridae”, show the weakest PPIs compared to their peers in the Picornaviridae and Dicistroviridae families. It is notable that in Secoviridae, although there are only two CPs (large and small), the large CP is composed of two beta‐barrel domains that occupy the positions of VP2 and VP3 of Picornaviridae, while the small subunit occupies the position of VP1, thereby resulting in *pT* = 3 architecture (Lin et al., 1999).

### 2.5 *T* = 4 capsids

**FIGURE 6:**
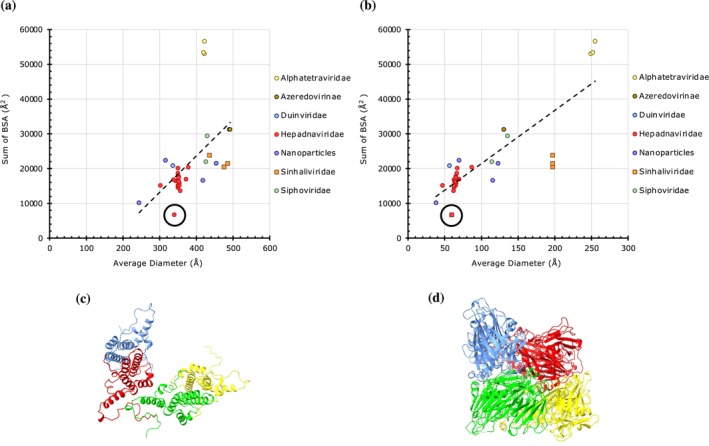
The magnitude of protein–protein interactions in *T* = 4 capsids. (a) A plot of cumulative sums of BSAs at the unique CP interfaces as a function of average diameter in *T* = 4 capsids (*n* = 38). Capsids are grouped by family as indicated in the legend on the right. The encircled outlier belongs to a hepadnavirus capsid assembled from the fused CP dimers (PDB: 7ZQ8). (b) A plot of cumulative sums of BSAs as a function of aggregate molecular weight of CPs in the asymmetric units in *T* = 4 capsids (*n* = 38). (c) Arrangement of CPs in the IAU of a Hepadnaviridae capsid of Hepatitis B virus (PDB: 6HTX) that displays moderate PPIs (cumulative BSAs: 17,700 Å^2^). (d) Arrangement of CPs in the IAU of an Alphatetraviridae capsid of Nudaurelia capensis omega virus (PDB: 1OHF) that exhibits strong PPIs (cumulative BSAs: 53,045 Å^2^).

*T* = 4 capsids (*h* = 2, *k* = 0) contain a total of 240 subunits, with four copies of a CP occupying an IAU. Analysis of 38 capsids from seven viral families suggests that their diameters range from 244 to 492 Å, while the BSAs vary between 6678 and 56,588 Å^2^ (Figure 6). Most families follow a positive correlation of BSAs as a function of diameters, as indicated by the trendline, with the exception of Alphatetraviridae and Sinhaliviridae. The capsids that show weaker interactions belong to the Sinhaliviridae (Chen et al., 2023) and one from Hepadnaviridae (Figure 6a,b). Interestingly, the significant outlier of Hepadnaviridae (PDB: 7ZQ8) (Kingston et al., 2023) (Figure 6a, encircled) is assembled from the two fused HBV CP dimers instead of four HBV CPs, which results in the loss of contribution from two significant dimer interfaces and hence is the reason for lower BSAs compared to their counterparts in the family. Moreover, notably, three *T* = 4 capsids from the Siphoviridae, a family that natively forms *T* = 7*l* capsids, closely follow the trendline. There are also three *T* = 4 nanoparticles that exhibit scattered BSAs and diameters.

In Alphatetraviridae, the CPs are considerably larger (~580 a.a.) compared to others (e.g., Hepadnaviridae CP is ~185 a.a.), thereby resulting in larger subunit interfaces and higher BSAs (Figure 6b,d) (Helgstrand et al., 2004). Notably, for the same reasons discussed for excluding the *T* = 3 members of Flaviviridae, we did not include the envelope glycoprotein shells of the Togaviridae family despite being classified as having *T* = 4 architecture in this study. The envelope glycoprotein subunits of Togaviridae have a transmembrane linker region that extends from the nucleocapsid protein on the inside connecting to the envelope glycoprotein layer on the exterior (Wang et al., 2022). Thus, each subunit of Togaviridae has three different chains, resulting in a total of 12 chains in an IAU (Figure S6). These additional chains add more interfaces, which in turn inflate the BSAs for a given capsid diameter, and therefore they were excluded from this study.

### 2.6 *T* = 7 capsids

**FIGURE 7:**
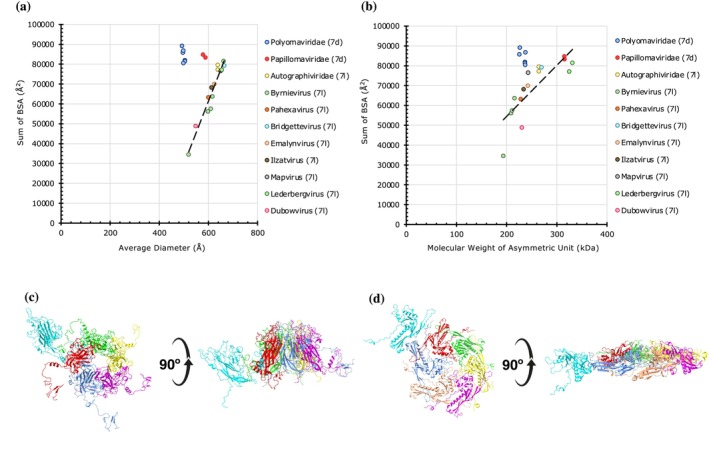
The extent of protein–protein interactions in *T* = 7*d* and *T* = 7*l* capsids. (a) A plot of cumulative sums of BSAs at the unique CP interfaces as a function of average diameter in *T* = 7*d* capsids (*n* = 8) and *T* = 7*l* capsids (*n* = 14). Capsids are grouped by family as indicated in the legend on the right. (b) A plot of cumulative sums of BSAs as a function of aggregate molecular weight of CPs in the asymmetric units in *T* = 7*d* capsids (*n* = 8) and *T* = 7*l* capsids (*n* = 14). Some of the *T* = 7*l* capsid outliers are structural assembly intermediates. (c) Arrangement of CPs in the IAU of a *T* = 7*d* capsid, Murine polyomavirus (PDB: 1SID) (cumulative BSAs: 81,873 Å^2^). Top and side views are provided. (d) Arrangement of CPs in the IAU of a *T* = 7*l* capsid, Bacteriophage HK97 mature empty capsid (PDB:1OHG) (cumulative BSAs: 63,611 Å^2^).

There are two types of *T* = 7 capsids with opposite handedness, *T* = 7*d* (*dextro; h = 1, k = 2*) and *T* = 7*l* (*levo; h = 2, k = 1*). Of the 22 capsids analyzed, eight of them display *T* = 7*d* architecture and belong to the families of Papillomaviridae and Polyomaviridae (Figure 7c) (Goetschius et al., 2021; Stehle & Harrison, 1996). These *T* = 7*d* capsids are entirely made up of CP pentamers. In other words, the 60 [(*T* − 1) × 10 = 60] hexavalent positions that exist in a *T* = 7 capsid are also occupied by the CP pentamers. Of note, all the capsids that are known to display *T* = 7*d* capsid architecture to date are composed of only the subunit pentamers and, as a result, contain only six subunits in the IAU. Therefore, the polyomavirus‐like (*T* = 7*d*) capsids are composed of 360 (6 × 60) subunits instead of the conventional 420 (7 × 60) subunits expected of a *T* = 7 capsid. However, the *T* = 7*l* capsids do contain 12 pentamers and 60 hexamers, thereby composed of 420 subunits (Figure 7d) (Helgstrand et al., 2003; Hryc et al., 2017). Of the eight *T* = 7*d* capsids analyzed, the diameters range from 492 to 588 Å and the BSAs from 80,410 to 89,097 Å^2^, while the *T* = 7*l* capsids (*n* = 14) display diameters that vary from 520 to 664 Å and the BSAs from 34,520 to 81,511 Å^2^ (Figure 7a,b). Interestingly, the maximum BSAs for both capsid types are in a similar range despite the T = 7*l* capsids being larger and containing one more CP in the IAU and 60 more in an entire capsid. Of note, many *T* = 7*l* capsids that are now listed as “unclassified” previously belonged to Podoviridae and Siphoviridae and show a wide range of BSAs. Some of the lower BSAs observed in *T* = 7*l* capsids belong to various maturation intermediates (e.g., prohead vs. head), which are less stable than matured capsids (Gan et al., 2006).

Moreover, the *T* = 7*d* capsids show strong PPIs, with BSAs falling between 80,000 and 90,000 Å^2^ (Figure 7a), for relatively smaller capsid diameters when compared to their *T* = 7*l* counterparts (Figure 7c). In addition to strong intra‐capsomer interactions (e.g., ~5790 Å^2^), the polyoma‐like *T* = 7*d* capsids display much stronger inter‐capsomer interactions, particularly at the icosahedral 2‐fold and quasi 2‐fold interfaces (e.g., ~9486 Å^2^) relative to the *T* = 7*l* capsids (Figure 7c,d). Separately, there is also a large spread of BSAs for *T* = 7*l* capsids (Figure 7a,b).

### 2.7 *T* = 9 capsids

**FIGURE 8:**
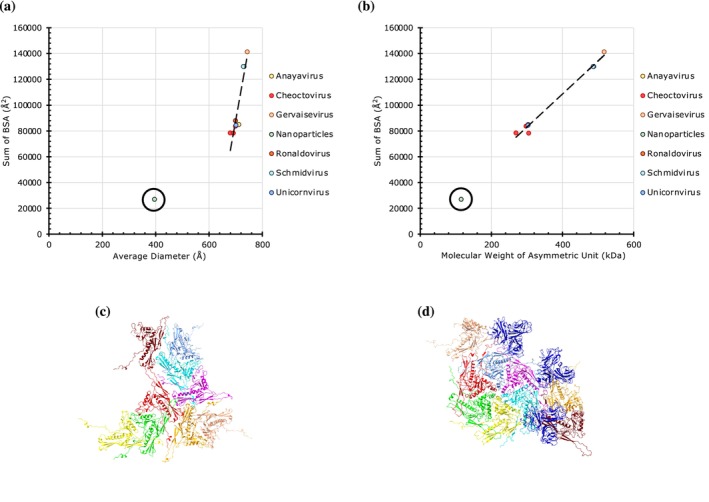
The magnitude of protein–protein interactions in *T* = 9 capsids. (a) A plot of cumulative sums of BSAs at the unique CP interfaces as a function of average diameter in *T* = 9 capsids (*n* = 10). Here we grouped the capsids by their genus as the majority of them belong to a yet‐to‐be‐classified family and indicated them in the legend on the right. The encircled outlier belongs to a nanoparticle derived from a bacterial microcompartment (PDB: 6MZX). (b) A plot of cumulative sums of BSAs as a function of aggregate molecular weight of CPs in the asymmetric units in *T* = 9 capsids (*n* = 10). (c) Arrangement of CPs in the IAU of Gordonia Phage (PDB: 8EB4) (cumulative BSAs: 87,827 Å^2^). (d) Arrangement of CPs in the IAU of Ralstonia phage capsid (PDB: 8H89) (cumulative BSAs: 141,629 Å^2^).

Adhering to the convention, all the ten *T* = 9 capsids (*h = 3, k = 0*) from four families analyzed contain nine CPs in the IAU and are composed of 540 subunits in a capsid with some variations (Table S1). The capsid diameters ranged from 396 to 744 Å and the BSAs from 26,803 to 141,629 Å^2^ (Figure 8). Notably, however, a nanoparticle derived from bacterial microcompartment (PDB: 6MZX) is a significant outlier that shows a smaller diameter (~400 Å) with correspondingly lower BSAs and has been classified to have *pseudo*‐*T* = 9 architecture (Greber et al., 2019). Upon further analysis, the CPs involved in forming this nanoparticle are composed of, on average, ~100 a.a., whereas the CPs of other native *T* = 9 capsids (see below) on average contain ~325 a.a. (Podgorski et al., 2023). This difference in the size of the CPs is responsible for the correspondingly lower BSAs observed in the nanoparticle.

As opposed to the native *T* = 9 capsids with diameters around 700 Å, three capsids from genera—Schmidvirus and Gervaisevirus exhibit slightly larger diameters (730–745 Å), but significantly higher BSAs (Figure 8a). In the latter capsids, in addition to having nine copies of a major CP in an IAU (540 per particle) forming a standard *T* = 9 chassis, the latter capsids have an extra nine copies of a small cement protein on their surface, a feature that is not found in other *T* = 9 capsids (Figure 8c,d) (Kamiya et al., 2022; Zheng et al., 2022). Even though the cement proteins did not raise the average capsid diameter significantly, they are responsible for a nearly 2× increase in the BSAs compared to the simpler *T* = 9 capsids, as it doubles the number of CPs and the interfaces.

## 3 CONCLUSIONS

While BSA is a simple and widely used metric for evaluating PPIs, it does not fully capture the energetic landscape of interactions involving hydrogen bonds, salt bridges, and hydrophobicity across the interfaces. However, BSA remains a useful proxy for representing the extent of PPIs. This study on evaluating the BSAs across a variety of quasi‐equivalent NEV capsids assembled from multiple copies of a single type of CP subunit or protomers has provided new insights into the variation of PPIs as a function of the molecular weight of the CP, capsid architecture, and size.

Overall, the natively forming viral capsids with *T* = 1, *T* = 3, and *T* = 4 icosahedral architectures exhibit a positive correlation between the capsid size (diameter) and strength (BSAs) with a few exceptions. It is notable that the non‐native and engineered (synthetic) capsids show relatively weak PPIs (Figures 2a and 4a). This is understandable given the need for the native (viral) capsids to be strong to survive the challenging environments, particularly outside of their hosts. Interestingly, the *pseudo*‐*T* = 3 (*pT* = 3) capsids, the majority belonging to the Picornaviridae family, are of one size of ~320 Å in diameter except for the viruses from Iflaviridae, which are slightly larger (360–380 Å) because of the additional decorations on the protomer subunits. Significantly, however, the *pT* = 3 capsids separate into two groups—full capsids and the altered capsids lacking the VP4 polypeptide, which show strong and weak PPIs, respectively (Figure 5a). The loss of PPIs observed in the altered capsids reflects biological/functional necessity for releasing the viral genome of Picornaviruses. Hence, BSAs provide a way to capture the variance in PPIs during the capsid metamorphosis in the course of the viral lifecycle. Similar structural changes are observed between prohead‐II and head‐II capsids of HK97 (Gertsman et al., 2009). The compact and expanded forms of CCMV (*T* = 3) capsids could be readily discerned by the variance in their BSAs, 16,019 and 7932 Å^2^, respectively. The expanded form has been implicated in the RNA release (Harder et al., 2023). The loss of PPIs observed in the expanded form is consistent with the results observed using atomic force microscopy (AFM) studies on CCMV (Wilts et al., 2015).

The “*T* = 2” capsids also show a positive correlation of BSAs versus diameters and MWs with the exception of the capsids from Picobirnaviridae that show exceptionally strong PPIs (Figure 3a,b). When it comes to *T* = 7 capsids, the *T* = 7*d* and *T* = 7*l* capsid architectures with opposite handedness cluster differently, where Polyomavirus‐like capsids (*T* = 7*d*) show relatively stronger PPIs compared to their *T* = 7*l* counterparts from Siphoviridae and Podoviridae families according to old taxonomy classification (Figure 7a,b). This difference in PPIs is particularly interesting because *T* = 7*d* capsids are made of 60 fewer subunits yet stronger than their *T* = 7*l* counterparts composed of 420 subunits. This is because, compared to the *T* = *7d* capsids, whose subunits exhibit “globular” shape (Figure 7c), the subunits of *T* = *7l* capsids display thin and “plate” like shape similar to the subunits of “*T* = 2” capsids of Totiviridae (Figures 7d and 3d,e). This results in relatively higher SASA (solvent accessible surface area) versus lower BSA in *T* = *7l* capsids compared to *T* = *7d* capsids.

**FIGURE 9:**
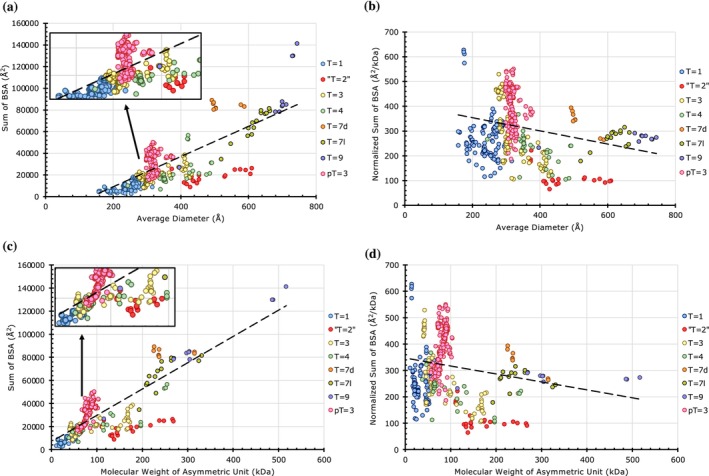
Combined analysis of protein–protein interactions in all the capsids considered in this study. (a) A plot of cumulative sums of BSAs as a function of average diameter in all the capsids considered in the data set (*n* = 634). Capsids are grouped by their *T*‐number. Note that the “*T* = 2” and the decorated *T* = 9 capsids cluster distinctly as the significant outliers. (b) A plot of per capsid normalized BSAs as a function of average capsid diameters. Normalized BSA of a capsid = summed BSA in an IAU of a capsid/total MW (kDa) of all CPs in the IAU. While the *T* = 1 Fiersviridae and Picornaviridae capsids show high normalized BSAs, the “*T* = 2” capsids show low normalized BSAs. (c) A plot of cumulative sums of BSAs as a function of aggregated molecular weight of the CPs in the asymmetric unit of all capsids in the data set (*n* = 634). (d) A plot of per capsid normalized BSAs as a function of aggregated molecular weight of the asymmetric unit.

**FIGURE 10:**
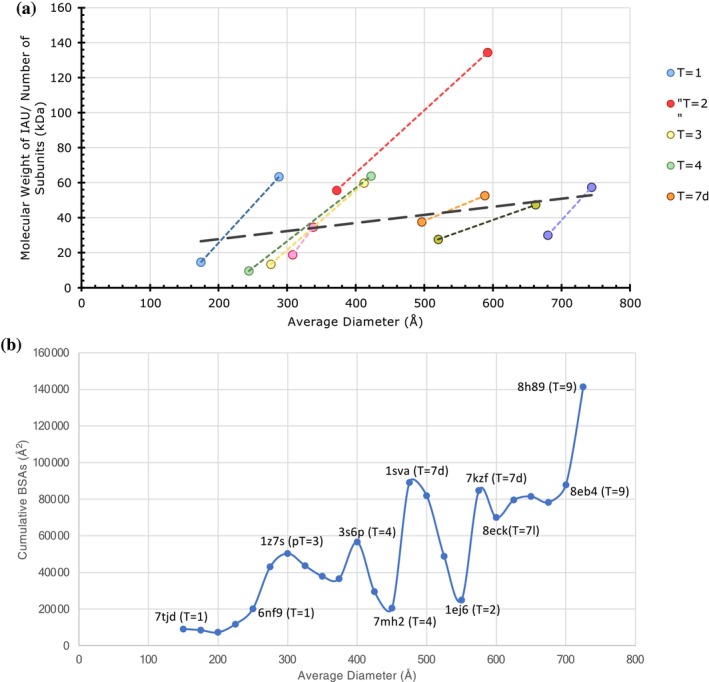
The observed ranges of capsid diameters and molecular weights (MW) of CPs forming various quasi‐equivalent capsids and the selection of strongest known capsids at various diameters. (a) A plot showing the extent of observed capsid diameters and the corresponding average MW of CPs of each quasi‐equivalent capsid identified by their *T*‐numbers shown in the legend. Note that even though there are overlaps of the range of diameters achieved by different *T*‐numbers, not surprisingly the larger size capsids are formed by the larger *T*‐numbered capsids for any given CP size. However, the “*T* = 2” capsids again are the outliers that overlap with the diameter ranges of *T* = 3, *T* = 4, and *T* = 7 capsids, but not with the *T* = 1 capsids. The trendline indicates that a CP in the MW range of 30–50 kDa can be used to build any quasi‐equivalent capsid exhibiting *T* = 1 to *T* = 9 architectures. (b) A plot of cumulative BSAs versus diameters identifying the strongest at various diameters at 25 Å intervals. A selected capsids identified by their PDB‐IDs and *T*‐numbers. More details of the data used to generate this plot can be found in Table S2.

When all the *T*‐number capsids considered in this study are grouped and analyzed together, a positive correlation appears between the BSAs and diameters (Figures 9 and S7). This trend is expected, as the higher T‐number capsids will have more CPs and interfaces; hence, higher BSAs. However, the “*T* = 2” capsids cluster distinctly as the outliers. With the exception of Picobirnaviridae, the weaker PPIs observed in the “*T* = 2” capsids may have a functional role in releasing the newly synthesized RNA transcripts of the dsRNA genome. Interestingly, normalizing the BSAs by the molecular weight of subunits occupying the IAU suggests that PPIs per CP subunit are rather constant and proportional to MW of the subunit across different *T*‐numbers and diameters (Figure 9b,d). Furthermore, estimating the range of capsid diameters and the corresponding CP molecular weights for various *T*‐numbered architectures suggests an overlap of capsid sizes between successive *T*‐number architectures (Figure 10). Notably, there is an overlap in the observed range of diameters of the capsids with the *T*‐numbers 1, 3, and 4, and separately between the *T* = 7 and *T* = 9 capsids (Figure 10). The sizes of *pseudo*‐*T* = 3 capsids appear to overlap with those of *T* = 3 and *T* = 4 capsids. Interestingly, the gap in the range of diameters between the *T* = 4 and *T* = 7 capsids is bridged by the “*T* = 2” capsids. Of note, there is no overlap in the range of diameters between the *T* = 1 and “*T* = 2” capsids. While the overall range of diameters of the quasi‐equivalent capsids analyzed in this study spanned between 158 and 744 Å, the molecular weight of the individual CPs occupying the IAU ranged between 14.5 and 124 kDa. Lastly, we identified the strongest capsids available at various diameters with intervals of 25 Å (Figure 10b, Table S2).

Furthermore, PPIs can be used to study the variability in stability/plasticity of capsid intermediates during virus maturation or genome release. Given that viral capsids are being used as gene and vaccine delivery vehicles and bioimaging reagents, the details of capsid characteristics in terms of the molecular weight of CP, capsid diameter, and strength discussed here are useful parameters to consider in choosing a capsid for specific applications. Future studies on viral capsids would entail the analysis of their assembly pathways and comparison of their surface characteristics.

## 4 METHODS

The 634 high‐resolution *uncomplexed* capsid structures (≤4 Å) from 39 viral families exhibiting *T*‐numbers ranging from 1 to 9 considered in the present study are shown in Table S1. The BSAs at the unique subunit interfaces were calculated using CHARMM as described previously (Carrillo‐Tripp et al., 2009; Reddy et al., 1998). Of note, the BSAs used in this study are ~2 times the contact areas that structural biologists usually estimate and report using the program PISA (Krissinel, 2015; Krissinel & Henrick, 2007). We chose to calculate BSAs this way because both interacting subunits lose surface area (SA) upon complex formation; BSA at the AB interface = (SA_A_ + SA_B_) − SA_(A+B)_. Furthermore, we calculated and used the sum of the BSAs for all interfaces involving the CPs that occupy an IAU that includes both the intra‐ and inter‐IAU interfaces for the comparisons done in this study. As previously noted, the magnitude of BSAs proportionally represents stronger or weaker PPIs in the respective capsids. The total BSAs at the unique CP subunit interfaces of different capsids and capsid diameters were obtained from VIPERdb (https://viperdb.org) (Carrillo‐Tripp et al., 2009; Montiel‐Garcia et al., 2021) and grouped according to the respective families and *T*‐numbers and plotted as the function of the average diameter of the respective capsids. Notably, the sum of BSAs used in this study roughly corresponds to 1/60th of the total BSAs observed in complete viral capsids because of the 60‐fold icosahedral symmetry. The structural illustrations reported in this study were generated using ChimeraX (Goddard et al., 2018; Meng et al., 2023; Pettersen et al., 2021). Scatter plots were generated using Microsoft Excel. Violin plots were generated using GraphPad Prism version 8.0.0 for Windows, GraphPad Software, San Diego, California.

## AUTHOR CONTRIBUTIONS

**Noah J. Zimmerman:** Conceptualization; investigation; methodology; formal analysis; writing – original draft; writing – review and editing; visualization. **Oscar Rojas Labra:** Software; formal analysis; methodology; data curation. **Vijay S. Reddy:** Conceptualization; methodology; data curation; validation; supervision; funding acquisition; visualization; project administration; writing – original draft; writing – review and editing.

## Supporting information


**Appendix S1:** Supporting information.

## Data Availability

The data that support the findings of this study are openly available in VIPERdb at https://viperdb.org.
