# Supplementary material for: Comparative analysis of the extent of protein–protein interactions in icosahedral viral capsids
Source: Protein Sci. 2025 Aug 25;34(9):e70257. doi: 10.1002/pro.70257 (PMC12375967; doi:10.1002/pro.70257)
Supplement: Supplementary file 1 — Appendix S1: Supporting information. [file PRO-34-e70257-s001.pdf]

Supplementary Material for

Comparative analysis of the extent of protein-protein interactions in icosahedral viral capsids

Noah J. Zimmerman[1,2], Oscar Rojas Labra[1], and Vijay S. Reddy[1,3*]

[1] *The Hormel Institute, University of Minnesota, Austin, MN 55912*

[2]*Department of Genetics, Cell Biology and Development, University of Minnesota, Minneapolis, MN 55455*

[3]*Department of Integrative Structural and Computational Biology, The Scripps Research Institute, La Jolla, CA 92037*

**Correspondence to: Vijay S. Reddy*

Address: 801 16th Ave NE, Austin, MN 55912

Office Phone: 507-437-9647

Fax Number:

E-mail: vsreddy@umn.edu

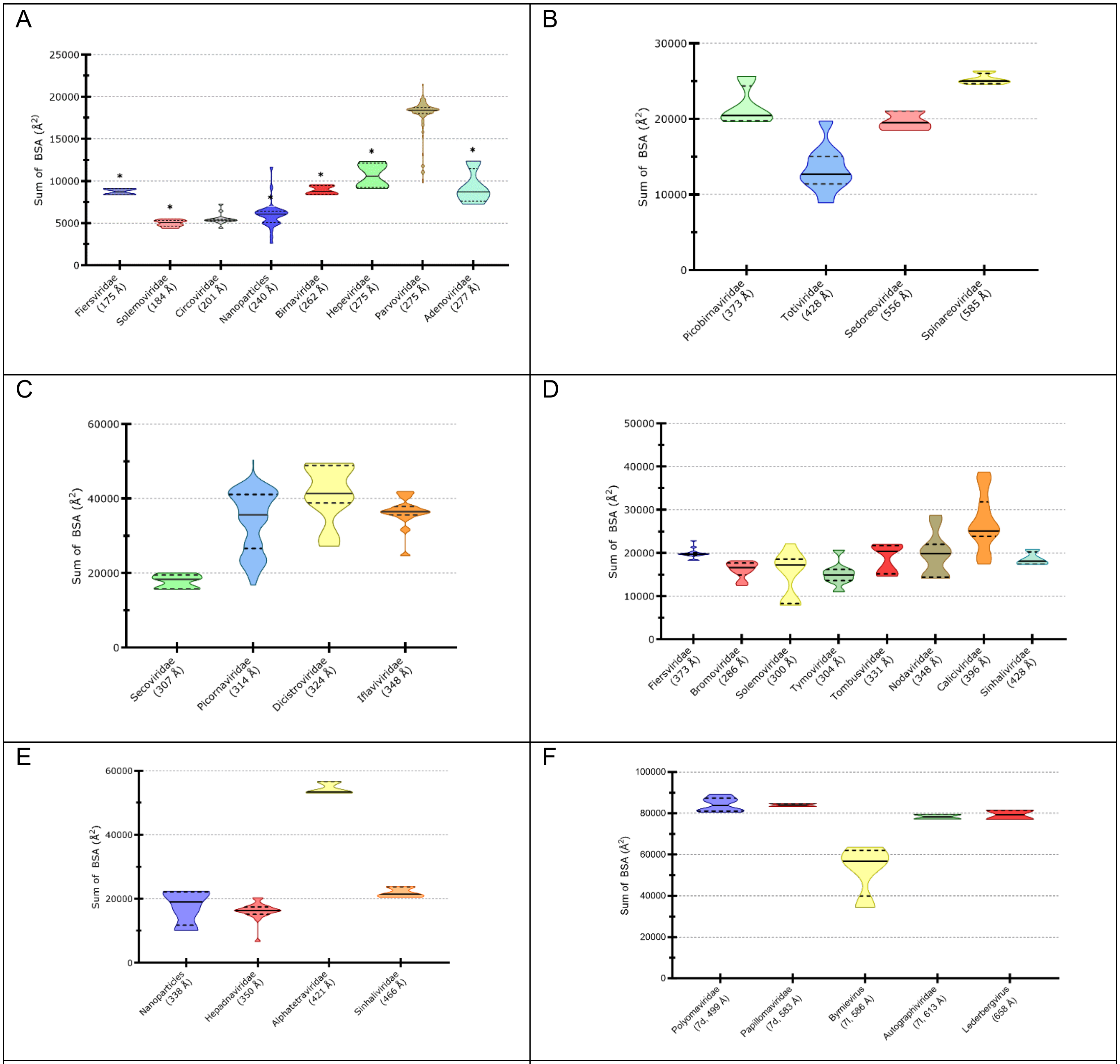

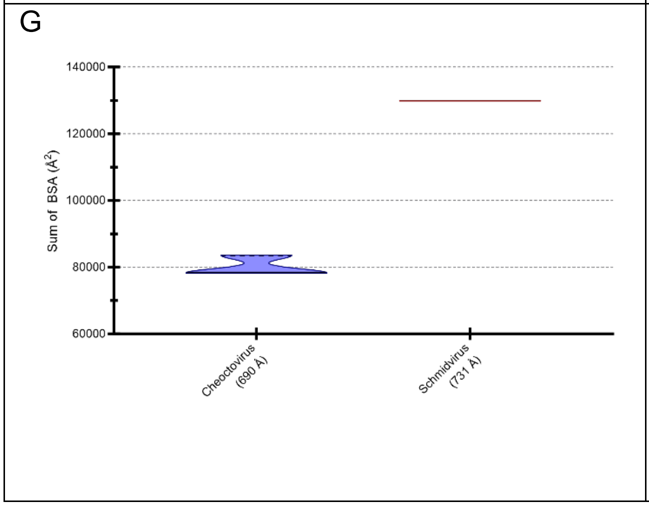

Figure S1. Violin plots showing the spread of BSAs of capsids grouped according to their T-number. The "violin" width correlates with the number of capsids with similar BSAs in the corresponding family. The solid line indicates the mean BSA, and the dashed lines represent quartiles. Average capsid dimeters (in Å) of each family are indicated along the X-axis. **(A)** Violin plot showing the spread of BSAs in T=1 capsids, grouped by families having at least four capsids. Asterisks indicate the families that contain non-native/synthetic capsids **(B)** Violin plot showing the spread of BSAs in "T=2" capsids grouped by the family. **(C)** Violin plot showing the spread of BSAs in *p*T=3 capsids. **(D)** Violin plot showing the spread of BSAs in T=3 capsids. **(E)** Violin plot showing the spread of BSAs in T=4 capsids. **(F)** Violin plot showing the spread of BSAs in T=7*l*/*d* capsids grouped by the family with at least two points. In the case of unclassified families, genus was used. **(G)** Violin plot showing the spread of BSAs in T=9 capsids with at least two points. In the case of unclassified families, genus name was used.

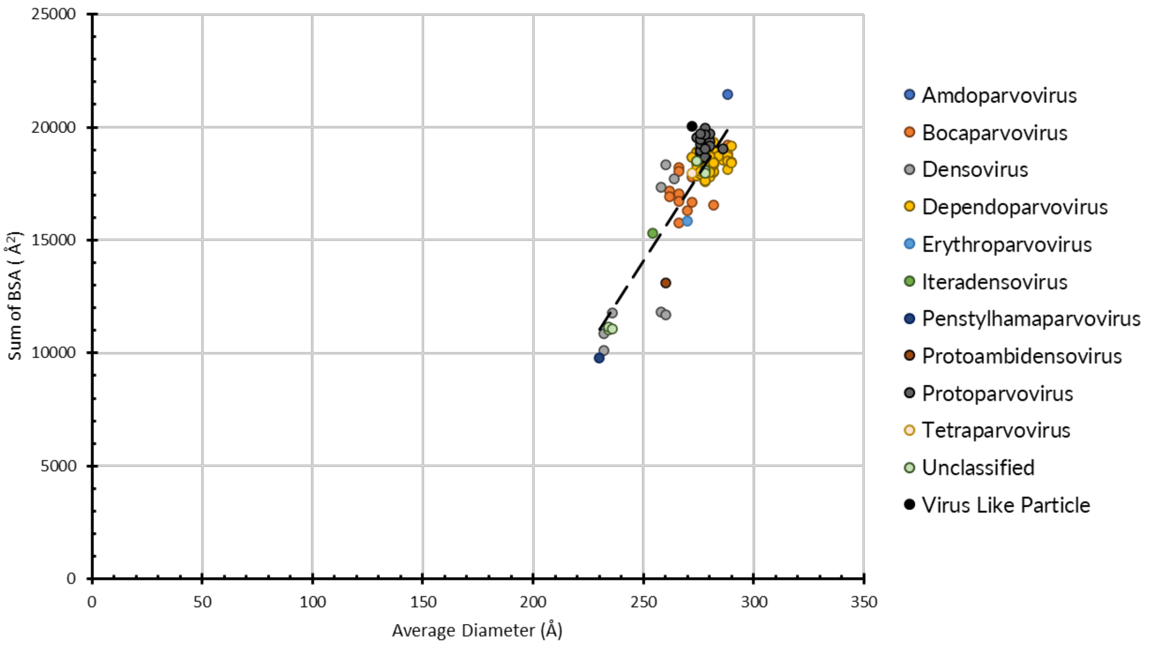

Figure S2. Protein-protein interactions in the T=1 capsids of Parvoviridae. The cumulative BSAs at the interfaces involving the CP occupying the IAU are plotted as a function of average diameter of the Parvoviridae capsids. The virus capsids are grouped by their genus as listed in the legend on the right.

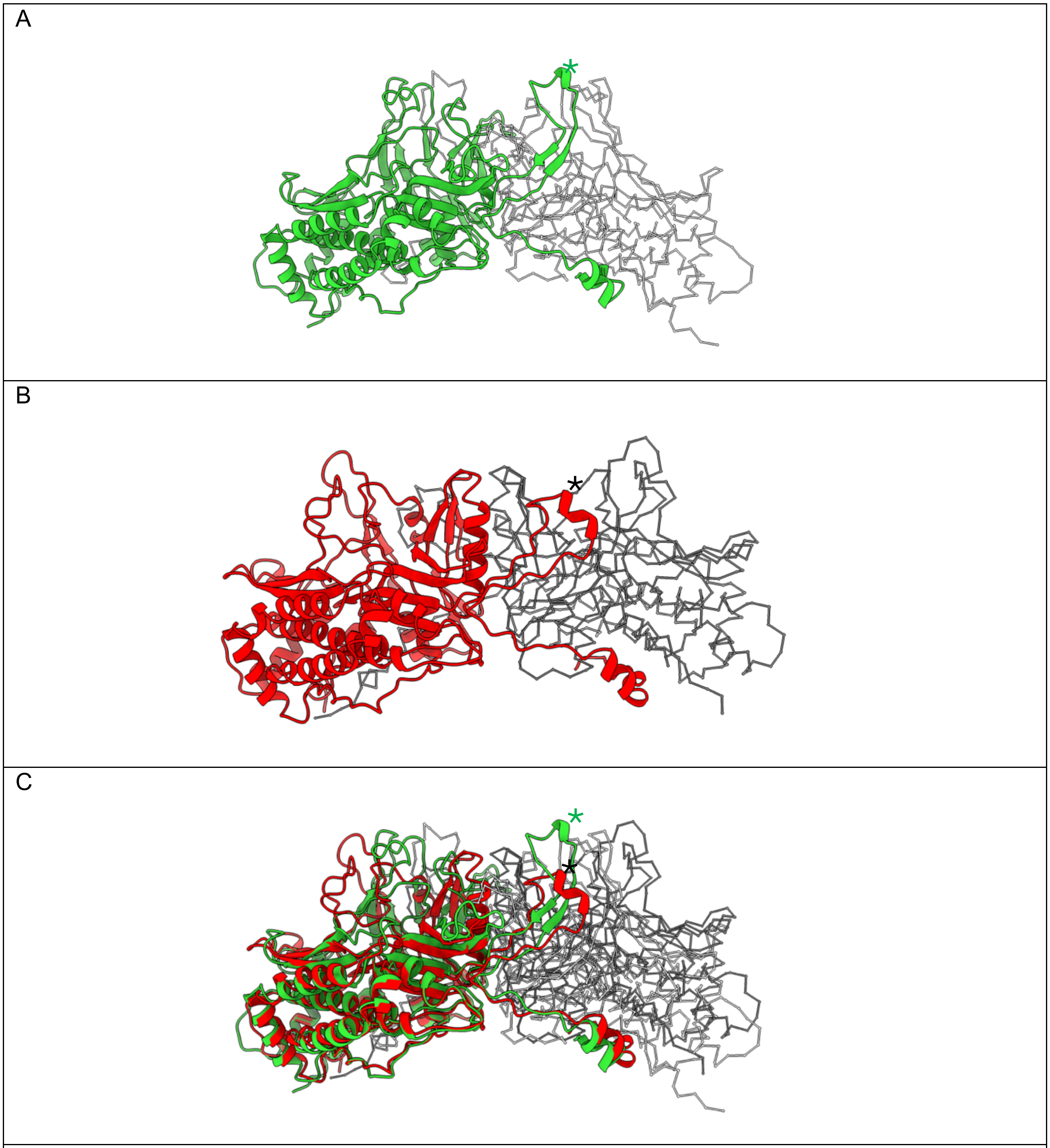

Figure S3. Structural comparison highlighting the differences in the CP (A-B) dimers that occupy the icosahedral asymmetric units (IAU) of “T=2” Picobirnaviridae capsids. **(A)** The A-B dimer of Rabbit picobirnavirus (PDB: 2vf1). The subunit-B of the rabbit picobirnavirus is shown in green and its partner subunit-A is shown in stick representation (light gray) for clarity. The prominent N-terminal surface loop (extension) is identified by a green asterisk. **(B)** The A-B dimer of Human picobirnavirus (PDB: 6z8e). The subunit-B of the human picobirnavirus is shown in red and its partner subunit-A is shown in stick representation (dark gray). The equivalent (less prominent) N-terminal surface extension is identified by a black asterisk. **(C)** Superposition of the A-B dimers. The B-subunits were used for the superposition of the AB-dimers.

A

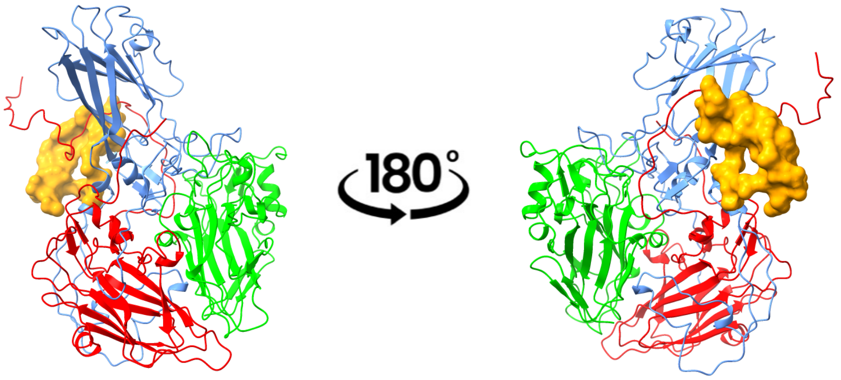

Full Picornaviridae capsid protomer/IAU (PDB: 1FPN); Sum of BSAs: 35,488 Å^2

B

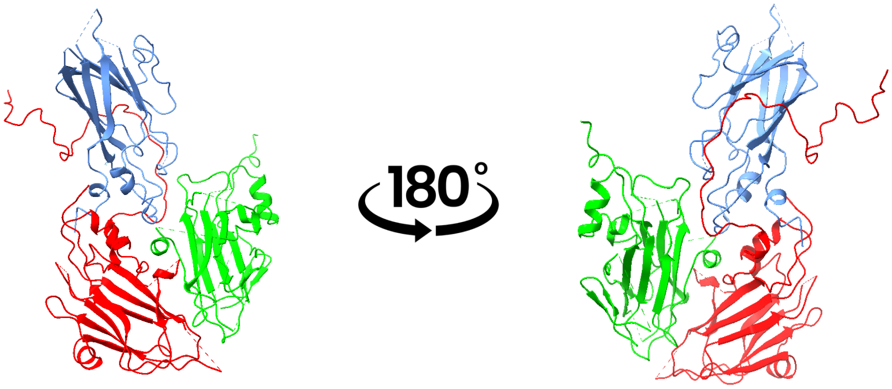

Altered Picornaviridae capsid protomer/IAU (PDB: 7NUM); Sum of BSAs: 16,733 Å^2

Figure S4. Comparison of protomers (IAUs) of Full and Altered capsids of Picornaviridae. **(A)** Protomer of a full Picornaviridae capsid (PDB: 1FPN). VP4 is represented in orange surface representation. Note that the full capsids exhibit strong PPIs. Top (left) and bottom (right) views are shown. **(B)** Protomer of an altered Picornaviridae capsid (PDB: 7NUM). Of note, altered capsids lack VP4 chain, which results in overall weaker PPIs in the altered capsids. Top (left) and bottom (right) views are illustrated.

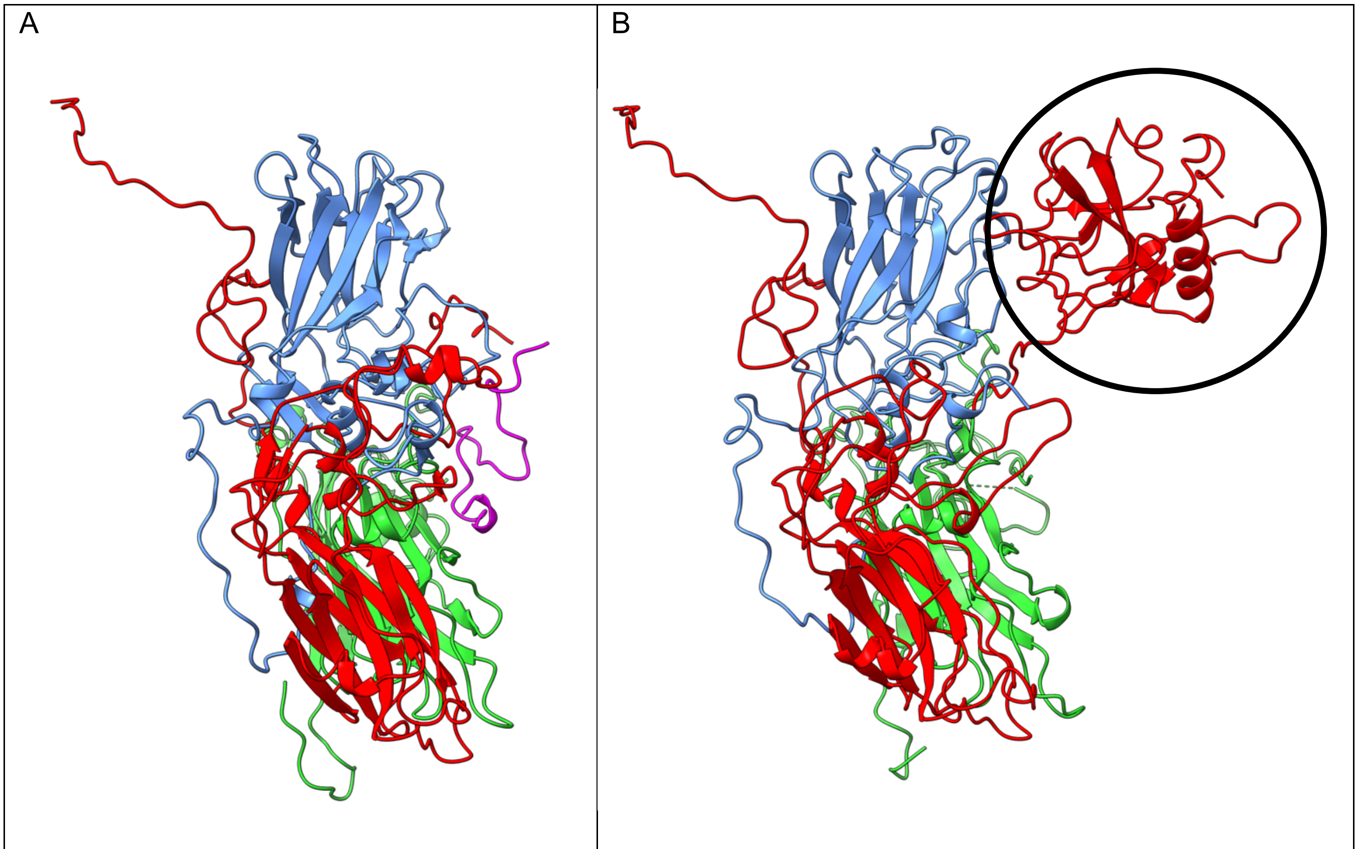

Sacbrood Honeybee Virus (PDB: 5LSF); Capsid diameter: 316 Å

Slow Bee Paralysis Virus (PDB: 5J96); Capsid diameter: 374 Å

Figure S5. Comparison of Iflaviridae protomers (icosahedral asymmetric units). **(A)** A side view of the protomer of Sacbrood honeybee virus (SBV) with a minor capsid protein (MiCP) is shown in magenta attached to VP2 (green) on the virion surface. **(B)** A side view of the protomer of an Iflaviridae, Slow bee paralysis virus (SBPV) with a protruding domain (encircled) attached to VP3 subunit that increases the average diameter of the capsid. While SBV (shown in panel-A) has typical diameter of a picornavirus (316Å), SBPV (panel-B) displays an enlarged diameter of 374Å.

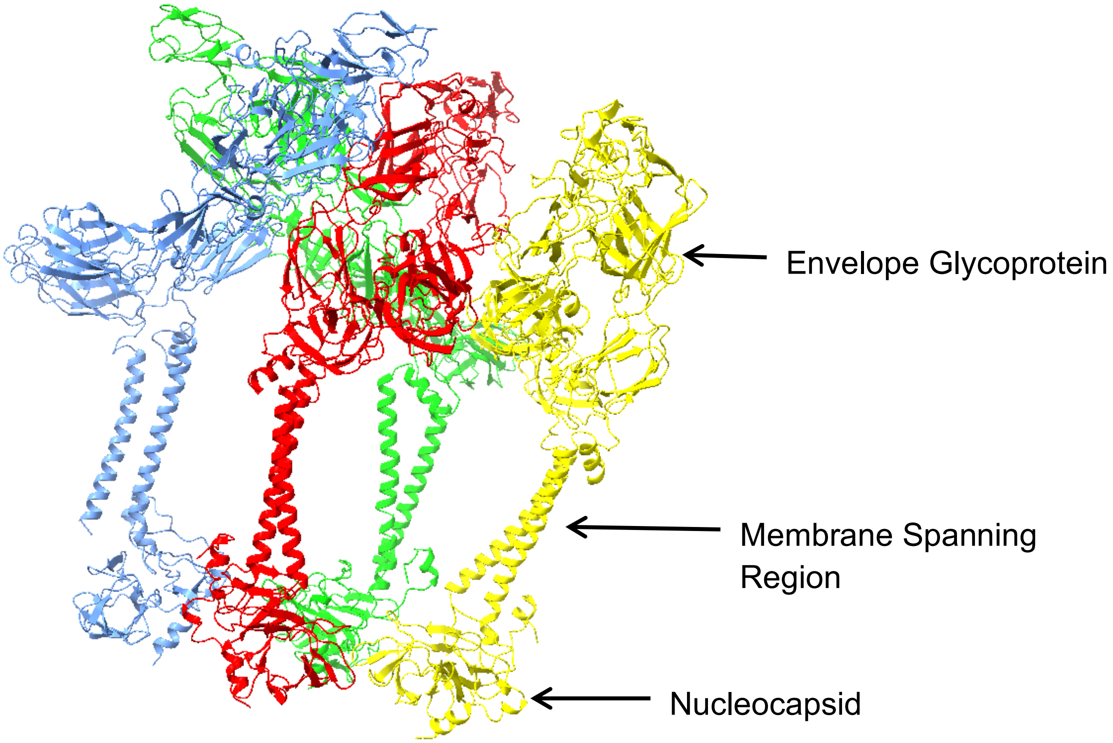

Figure S6. A side view of the IAU of a T=4 Alphavirus of Togaviridae family (PDB: 7wc2) composed of four chains, each shown in different colors. Each chain consists of 3 annotated regions: Nucleocapsid, Membrane Spanning Region and Envelope Glycoprotein.

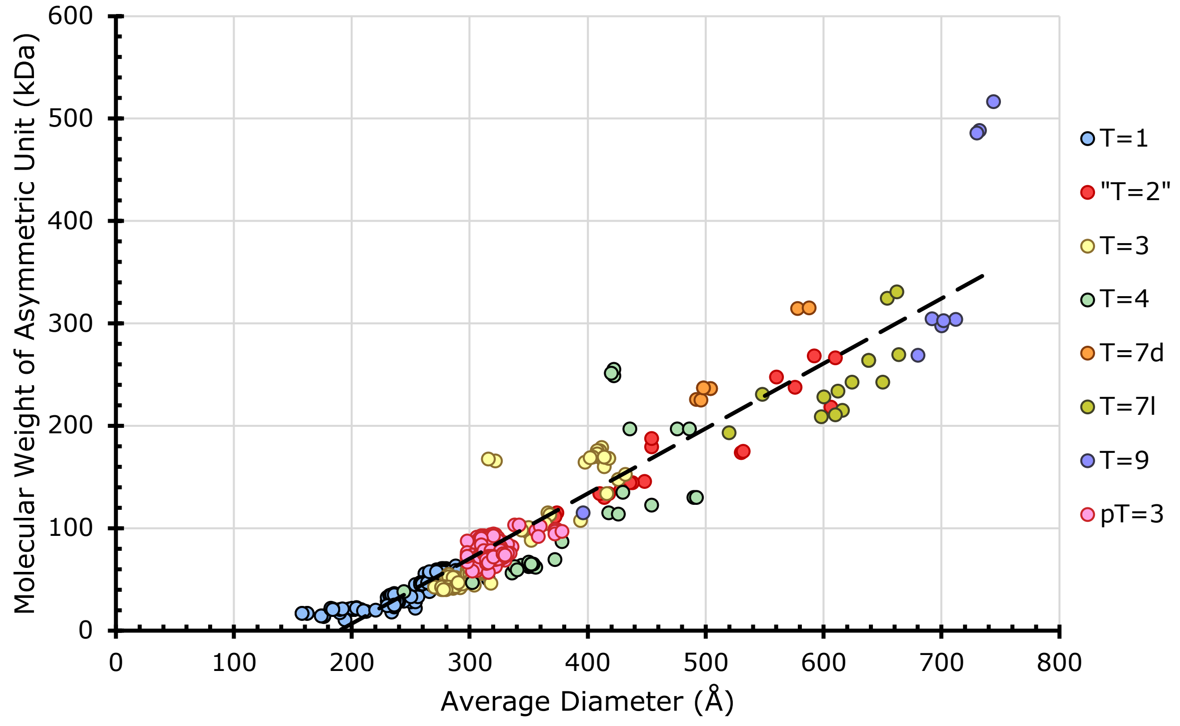

Figure S7. A plot of aggregate molecular weight of CPs in the IAU vs. corresponding capsid diameter for all the capsids (N=634) included in the study. The T-numbers of the capsids are identified in the legend accordingly.

Table S1. Information about all viral and synthetic capsids used in the study.

T=1 Capsids (n=213)		
**Family**	**Number of Capsids**	**PDB-IDs**
Adenoviridae	4	1x9p, 2c6s, 4ar2, 6hcr
Birnaviridae	5	1wcd, 2df7, 2gsy, 3fbm, 3ide
Bromoviridae	3	1yc6, 7epp, amv
Caliciviridae	1	6ouc
Circoviridae	16	3jci, 3r0r, 5j36, 5zju, 6dzu, 6e2r, 6e2x, 6e2z, 6e30, 6e32, 6e34, 6e39, 6ola, 6rpk, 6rpl, 6rpo
Fiersviridae (Leviviridae)*	4	4zor, 7tjd, 7tje, 7tjg
Geminiviridae	1	8uh4
Hepeviridae	4	2ztn, 2zzq, 3hag, 6lat
Microviridae	2	9ffg, 9ffh
Nanoparticles	31	6i9g, 6wkv, 7b3y, 7bcv, 7boj, 7kq5, 7lii, 7lij, 7lik, 7lil, 7lim, 7lis, 7lit, 7mu1, 7odw, 7p1t, 7phm, 7s21, 7sge, 7x7m, 8piu, 8pvj, 8pys, 8qbx, 8rb3, 8t6r, 8u50, 8uyo, 8xb8, 8yt4, 9ez8
Nodaviridae	3	4rft, 5yl1, 6ab5
Papillomaviridae	1	1dzl
Parvoviridae	130	1c8d, 1c8e, 1c8f, 1c8g, 1c8h, 1dnv, 1fpv, 1k3v, 1lp3, 1p5y, 1s58, 1z14, 1z1c, 2cas, 2g8g, 2qa0, 3kic, 3kie, 3n7x, 3ng9, 3ntt, 3oah, 3p0s,3ra2, 3ra4, 3ra8, 3ra9, 3raa, 3ux1, 4g0r, 4gbt, 4iov, 4qc8, 4qyk, 4rso, 4zpy, 5ipi, 5ipk, 5uf6, 5urf, 5us7, 5us9, 6b9q, 6bwx, 6bx0, 6bx1, 6cbe, 6e9d, 6ih9, 6jcr, 6jct, 6nf9, 6o9r, 6pwa, 6u0r, 6u0v, 6u20, 6u2v, 6u3q, 6u95, 6ubm, 6v10, 6v12, 6v1g, 6v1t, 6v1z, 6wft, 6wfu, 6wh3, 6wh7, 6x2i, 6x2k, 7jot, 7kfr, 7kp3, 7l0u, 7l0v, 7l0w, 7l0x, 7l0y, 7l5q, 7l5u, 7l6a, 7l6b, 7l6e, 7l6f, 7l6h, 7l6i, 7lnk, 7ltm, 7m3o, 7mt0, 7mtg, 7mtp, 7mtw, 7na6, 7rk8, 7rk9, 7rl1, 7rwl, 7rwt, 7thr, 7ti4, 7u94, 7u95, 7u96, 7u97, 7ud4, 7wjw, 7wqo, 8a9u, 8ep2, 8ep9, 8erk, 8eu5, 8eu6, 8eu7, 8fq4, 8jre, 8t9c, 8t9e, 8t9x, 8ta7, 8tex, 8tey, 8tje, 8tu0, 8tu1, 8tu2, 8xeg
Retroviridae	2	6ssj, 7no0
Solemoviridae	6	1vak, 1vb2, 1vb4, 1x36, 4y4y, 4y5z
**T=2 Capsids (n=21)**		
**Family**	**Number of Capsids**	**PDB IDs**
Picobirnaviridae	4	2vf1, 6z8d, 6z8e, 6z8f
Totiviridae	10	1m1c, 6y83, 7m12, 7ns2, 7qwx, 7qwz, 7z90, 8pe4, 8r0f, 8r0g
Sedoreoviridae	3	1uf2, 3gzu, 3kz4
Spinareoviridae	4	1ej6, 3iz3, 3jay, 6djy
**T=3 Capsids (n=89)**		
**Family**	**Number of Capsids**	**PDB IDs**
Bromoviridae	10	1cwp, 1f15, 1js9, 1laj, 1za7, 3j7l, 3j7m, 3j7n, 7pe1, 7pe2
Caliciviridae	18	1ihm, 2gh8, 3m8l, 6gsh, 6iuk, 6otf, 6p4l, 7bjp, 7dod, 7mry,

		7n6y, 7n7f, 8vgr, 8vjr, 8vjs, 9cve, 9cvf, 9cvg
Duinviridae	1	8twc
Fiersviridae (Leviviridae)*	19	1bmx, 1dwn, 1fr5, 1frs, 1gav, 1mst, 1mva, 1mvb, 1qbe, 2ms2, 2w47, 2w4z, 2vf9, 5kip, 5vly, 6rrs, 7eoy, 7tjm, 7zqa
Nodaviridae	11	4nwv, 4nww, 6ab6, 6h2b, 6jjc, 6jjd, 6rj0, 8yf6, 8yf7, 8yf8, 8yf9
Sinhaliviridae	4	7xpa, 7xpd, 7xpf, 7xpg
Solemoviridae (Sobemovirus)*	9	1f2n, 1ng0, 1smv, 2izw, 2vq0, 4sbv, 6rtk, 6sco, 9fhp
Tombusviridae	9	1c8n, 1opo, 2tbv, 2zah, 4llf, 4v99, 6mrl, 6mrm, 6scl
Tymoviridae	8	1auy, 1e57, 1qjz, 1w39, 2fz1, 2wws, 7sqy, 7sqz
**Pseudo T=3 Capsids (n=241)**		
**Family**	**Number of Capsids**	**PDB IDs**
Dicistroviridae	11	1b35, 3nap, 5cdc, 5l70, 5lwg, 5lwi, 5mqc, 6iic, 7bc3, 7bg8, 7bgk
Iflaviviridae	15	5g52, 5j96, 5j98, 5l7q, 5l8q, 5lk7, 5lk8, 5lsf, 5mup, 5mv5, 5mv6, 5oyp, 6egv, 6eiw, 6f5j
Picornaviridae	208	1al2, 1ar6, 1ar7, 1ar8, 1ar9, 1asj, 1aym, 1ayn, 1bbt, 1bev, 1cov, 1d4m, 1ev1, 1fmd, 1fod, 1fpn, 1h8t, 1hxs, 1k5m, 1mec, 1mqt, 1nd2, 1oop, 1pov, 1pvc, 1qqp, 1r1a, 1rhi, 1rmu, 1ruf, 1ruj, 1tme, 1tmf, 1z7s, 1zbe, 2mev, 2plv, 2rmu, 2wff, 2ws9, 2wzr, 2x5i, 3cji, 3jb4, 3jd7, 3tn9, 3vbf, 3vbh, 3vbo, 3vbr, 3vbs, 3vbu, 4aed, 4gh4, 4gmp, 4iv1, 4iv3, 4jgy, 4jgz, 4n43, 4n53, 4pdw, 4q4v, 4q4w, 4q4x, 4q4y, 4qpg, 4qpi, 4rhv, 4rqp, 4rr3, 4rs5, 4wm8, 4yvs, 4yvw, 4z92, 5ac9, 5aca, 5aoo, 5c4w, 5c8c, 5c9a, 5cfc, 5cfd, 5d8a, 5ddj, 5gka, 5jzg, 5k0u, 5mjv, 5ne4, 5ned, 5nej, 5o5b, 5osn, 5wte, 5wtf, 5xs4, 5xs5, 5yhq, 6acu, 6acw, 6acy, 6ads, 6adt, 6aj0, 6aj3, 6aks, 6akt, 6aku, 6crp, 6crr, 6crs, 6cru, 6cs3, 6cs4, 6cs5, 6cs6, 6csa, 6csg, 6csh, 6cv1, 6cv2, 6cv3, 6cv4, 6cv5, 6dij, 6gzv, 6hbg, 6hbh, 6hbj, 6iij, 6iio, 6iln, 6ilo, 6ilp, 6la3, 6la4, 6lap, 6lb1, 6lbo, 6lbq, 6lha, 6lhb, 6lhc, 6mzi, 6o06, 6rjf, 6sk6, 6sk7, 6smg, 6uh1, 6z6w, 6zms, 7bg6, 7bzn, 7bzo, 7c4t, 7c4w, 7c4y, 7c4z, 7c9s, 7c9t, 7c9u, 7c9x, 7c9y, 7c9z, 7dpf, 7eah, 7eai, 7eno, 7enp, 7nul, 7num, 7nuo, 7opx, 7ozi, 7qvx, 7qvy, 7qw9, 7t9p, 7thx, 7vxh, 7vxl, 7vxn, 7vy0, 7vy5, 7vy6, 7w17, 7wl3, 7xb2, 7xxg, 7xxj, 8anw, 8at5, 8aw6, 8axx, 8bqn, 8cxp, 8f7y, 8hi2, 8r5x, 9fjc, 9g0b
Secoviridae	7	1a6c, 1bmv, 1ny7, 1pgl, 1pgw, 2bfu, 7chk
**T=4 Capsids (n=38)**		
**Family**	**Number of Capsids**	**PDB IDs**
Alphatetraviridae	3	1ohf, 3s6p, 7anm
Azeredovirinae	2	8vd4, 8vd5
Duinviridae	1	8tw2
Hepadnaviridae	23	1qgt, 2g33, 3j2v, 6htx, 6hu4, 6hu7, 6ui6, 6ui7, 6vzp, 6ygh, 6ygi, 7abl, 7ep6, 7oco, 7ocw, 7od4, 7od6, 7od7, 7od8, 7oen, 7oev, 7oew, 7zq8
Nanoparticles	4	7a4j, 7ckc, 7mh2, 8rvj
Sinhaliviridae	3	7xgz, 7xpb, 7xpe
Siphoviridae	2	6b23, 7rwz
**T=7 Capsids (n=22)**		
**Family/ Genus if no family**	**Number of**	**PDB IDs**

	Capsids	
Papillomaviridae (T=*7d*)	2	7kzf, 7ryj
Polyomaviridae (T=*7d*)	6	1sid, 1sie, 1sva, 6esb, 6zlz, 6zml
Autographiviridae (T=*7l*)	2	3j7w, 3j7x
Bridgettevirus (T=*7l*)	1	8eci
Byrnievirus (T=*7l*)*	4	1ohg, 2fsy, 2ft1, 3e8k
Dubowvirus (T=*7l*)*	1	6b0x
Emanlynvirus (T=*7l*)	1	8eck
Ilzatvirus (T=*7l*)	1	8eco
Lederbergvirus (T=*7l*)*	2	5l35, 5uu5
Mapvirus (T=*7l*)*	1	8edu
Pahexavirus (T=*7l*)	1	3jb5
**T=9 Capsids (n=10)**		
**Family/ Genus if no family**	**Number of Capsids**	**PDB IDs**
Anayavirus*	1	8ec2
Cheoctovirus*	3	8e16, 8ec8, 8ecn
Gervaisevirus*	1	8h89
Nanoparticle	1	6mzx
Ronaldovirus*	1	8eb4
Schmidvirus*	2	7dn2, 7f2p
Unicornvirus*	1	8ecj

*The instances where the virus family was listed as unclassified, so their genus name is used instead

Table S2. Strongest capsids identified at various diameters at 25Å intervals*

Diameter Range	PDB ID	T- numbers	Sum BSA	IAU MW	MW of CP
150-174	7tjd	1	9105	14.52	14.52
175-199	4zor	1	8462	13.86	13.86
200-224	5j36	1	7237	22.66	22.66
225-249	6wh3	1	11772.1	36.74	36.74
250-274	6nf9	1	20063	59.51	59.51
275-299	7enp	pT3	43050	87.78	29.26
300-324	1z7s	pT3	50340	94	31.33
325-349	7vy5	pT3	43701	103	34.33
350-374	5mv5	pT3	37874	98.89	32.96
374-399	6f5j	pT3	36473	96.91	32.3
400-424	3s6p	4	56588	255.09	63.77
425-449	6b23	4	29404	135	33.75
450-474	7mh2	4	20449	197.12	49.28
475-499	1sva	7d	89097	226.27	37.71
500-524	1sid	7d	81872	236.61	39.43
525-549	6b0x	7l	48825	230.67	32.95
550-574	1ej6	2	24952	247.72	123.86
575-599	7kzf	7d	84754	615.04	102.5
600-624	8eck	7l	69973	242.55	34.65
625-649	3j7x	7l	79512.7	264.11	37.73
650-674	5uu5	7l	81511	331.1	47.3
675-699	8e16	9	78330	269.28	29.92
700-724	8eb4	9	87827	318	35.33
725-750	8h89	9	141269	517	57.44

*This data was used to generate Fig. 10B.
